# Effects of Mobile-Based Forest-Therapy Programs Using Urban Forests for Symptoms of Depressed Patients

**DOI:** 10.3390/healthcare11233039

**Published:** 2023-11-25

**Authors:** Poung-Sik Yeon, In-Ok Kim, Si-Nae Kang, Nee-Eun Lee, Ga-Yeon Kim, Ha-Rim Shim, Chung-Yeub Chung, Jung-Sok Lee, Jin-Young Jeon, Won-Sop Shin

**Affiliations:** 1 Department of Forest Sciences, Chungbuk National University, Cheongju 28644, Republic of Korea; well@chungbuk.ac.kr; 2 Graduated Department of Forest Therapy, Chungbuk National University, Cheongju 28644, Republic of Korea; inoya88@chungbuk.ac.kr (I.-O.K.); sinae375@chunguk.ac.kr (S.-N.K.); share1227@chunguk.ac.kr (N.-E.L.); yeon6520@chungbuk.ac.kr (G.-Y.K.); cacris@chungbuk.ac.kr (H.-R.S.); 3 Gwanghwamun Forest Psychiatric Clinic, Seoul 03156, Republic of Korea; 4 Maumlab, Seoul 03156, Republic of Korea; 5 Korea Forest Therapy Forum Incorporated Association, Cheongju 28644, Republic of Korea

**Keywords:** forest therapy, depression, urban forest, mobile healthcare

## Abstract

This study investigated the effect of mobile-based forest therapy programs on relieving depression to advance non-pharmaceutical treatments for patients with depression. The effects of depression, sleep quality, and physical symptoms were analyzed as measurement indicators to determine the effectiveness of symptom relief in patients with depression. This study used a randomized controlled experiment design. Participants were randomly assigned, and a total of 44 people participated, including 23 in the experimental group and 21 in the control group. The experimental group participated in a mobile-based forest therapy program (participating once a week) for six sessions. As a result of this study, depression patients who participated in the mobile-based forest therapy program conducted in urban forests showed a significant reduction in MADRS (from 21.48 ± 4.05 to 7.13 ± 7.00). In addition, PSQI (from 19.78 ± 7.69 to 14.48 ± 8.11) and PHQ-15 (from 9.87 ± 5.08 to 7.57 ± 5.03) were also found to significantly improve symptoms. This suggests that forest-therapy programs using mobile applications can be applied as non-pharmaceutical interventions to relieve symptoms in patients with depression.

## 1. Introduction

Depression is no longer considered a hard-to-see mental illness. According to the World Health Organization (WHO) [1], approximately 280 million people worldwide suffer from depression, of which adults account for approximately 5%. According to an Organization for Economic Cooperation and Development (OECD) report [2], 4 out of 10 Koreans suffer from depression, and the prevalence of depression is 36.8% (as of 2020), which is the highest among OECD countries. Public health measures, such as social distancing to prevent the spread of COVID-19 [3], have further increased the prevalence of depression worldwide, as social isolation, separation, and anxiety have formed [1,4,5,6,7,8,9].

Depression is not only about feeling depressed, but also physical symptoms such as lethargy, sleep disorders, somatic symptoms, and cognitive decline in the likes of memory and attention [10,11,12,13,14,15]. These symptoms make it difficult for depressed patients to continue their normal daily lives, and in severe cases, lead to suicidal incidents [16]. Despite the severity of depression, the benefits of mental health services were also limited during the COVID-19 period due to restrictions on the scope of individual activities. According to a mental health survey conducted in 2022 in South Korea [17], the depression risk group was 18.5%, higher than before the COVID-19 pandemic (2019 years, 3.2%), and awareness of mental health services was only 18.1%. The prolonged COVID-19 pandemic has accelerated the need to offer better solutions for medical access to depressive disorders and mental health services. As a result, there is interest in mental health services using easily accessible mobile devices to prevent and improve depression [4,18,19].

Currently, the market for mental health services using mobiles is expanding [20,21], and the number of mental-health-related applications exceeds 10,000 [22], resulting in quantitative growth. According to a survey by Sensor Tower [23], a data analysis company in the global mobile market, more than four million mental-health-related applications were downloaded in April 2020, when social distancing was lifted in the United States. This is an increase of 17.6% compared to January when the COVID-19 pandemic restricted activities. The main applications used to relieve depression involve a large proportion of activities such as meditation and mindfulness [24,25]. Studies have attempted to determine the effectiveness of mobile applications as they become increasingly utilized [26,27,28]. Arean et al. [27] compared the effects of three types of mental-health-management applications (cognitive control app, problem solving therapy app, information control) on depression relief for 12 weeks and found that cognitive and problem-solving applications had a positive effect on mood conditions. Ly et al. [28] reported that in subjects diagnosed with major depressive disorder they had a positive effect on depression and mindfulness because they allowed them to experience behavioral activation (BA) and mindfulness-based applications for 8 weeks.

However, very few applications provide evidence-based mental health services to date, even though conditions for therapeutic access using mobile devices have been created due to the COVID-19 pandemic and due to the development of information and communication technology [18,29,30]. A new proposal to apply evidence-based non-pharmaceutical interventions using mobile devices in patients with depression is to apply forest-therapy programs. Existing treatment approaches for patients with depression are based on drug treatment [31,32,33], but in recent years, the importance of applying various non-pharmaceutical therapies such as psychotherapy and cognitive behavior therapy along with drug therapy has increased [34,35,36,37,38]. Non-pharmaceutical therapy needs to alleviate barriers to treatment for patients with depression and to try new treatment methods for treatment prognosis.

Forests contain forest healing elements such as phytoncides, landscapes, natural sounds, and scents. The forest stimuli that humans receive when they experience forests directly or indirectly are psychologically, physiologically, and cognitively healing effects [39,40,41,42,43,44,45]. The program has been applied to patients with depression to prove its effectiveness using scientific approaches [46,47,48,49,50,51]. Woo et al. [47] investigated the effectiveness of forest healing programs in patients with major depression. The group of patients who participated in the forest healing program showed a significant improvement in depression compared to the group that received daily outpatient treatment. In the study by Rosa et al. [49], which meta-analyzed the effect of improving depression, the average effect size was 1.18 (95% CI: 0.86 to 1.50, *p* < 0.0001), indicating that forest therapy is a more effective short-term intermediary for preventing and treating adult depression. These findings suggest that the forest healing program developed to lower the level of depression in participants is worth using as a nonpharmaceutical intervention, emphasizing the importance of forest healing factors that affect the improvement of depression.

Previous studies have reported that depression has been alleviated through forest-therapy programs, but the existing forest-therapy programs were delivered with the guidance of forest healing instructors, and most of them were conducted for many people [46,47]. In addition, the effectiveness of the forest-therapy program has not been evaluated by psychiatrists despite progress in depression patients. In addition, the sites of forest-therapy programs conducted in previous studies were in places with excellent natural environments such as healing forests, the national center for forest therapy, and natural recreation forests, but they are far from their residences [51,52,53]. Research to verify the effectiveness of forest-therapy programs for patients with depression is still lacking, and more evidence is needed regarding the health benefits of forest-therapy programs as part of new mobile healthcare. As the main symptoms of patients with depression are lethargy and decreased activity, research is needed to lower their resistance to psychiatric treatment and to suggest practical measures utilizing urban forests with good living conditions and accessibility.

Therefore, this study attempted to investigate the effects of mobile-based forest-therapy programs conducted in urban forests on the depression state, sleep quality, and physical symptoms of patients with depression.

## 2. Materials and Methods

### 2.1. Study Design

This study used a randomized controlled experiment design. To ensure homogeneity between the groups, the study subjects were randomly allocated to an experimental group that participated in a mobile-based forest-therapy program and a control group that did not participate. A random-number generator was used for the random extraction. The study participants were randomly assigned numbers, the experimental group was assigned odd numbers, and the control group was assigned even numbers. The experimental group participated in the mobile-based forest-therapy program for six sessions (once a week), and the control group lived their daily lives under the regular treatment that they had previously received. Mobile-based forest-therapy programs were carried out from April to June 2023, and pre-testing was conducted in the first week (25–27 April) and last week (9–13 June) of the study period. The pre- and post-test were conducted through a mobile application (Metri) as a psychiatrist’s evaluation (MADRS) and a self-report questionnaire (PSQI, PHQ-15).

### 2.2. Participants

The participants in this study were selected from four psychiatric clinics in Seoul to target patients treated for depression. Participants were recruited for about a month (30 March to 24 April 2023); promotional posters in the hospital were posted, and pamphlets were placed to recruit them. The criteria for selecting the study participants were as follows. (a) Adults in their 20s or older who were proficient in smartphone use. (b) Adults with no motor disorders or other physical disorders who had difficulty participating in a two-hour outdoor activity program. (c) Adult diagnosed with mild depressive disorder under DSM-5 by a psychiatrist. There were 47 depressed patients who expressed their intention to participate in this study, but three people (experimental group: difficulty visiting other regions, control group: difficulty in participating in the study due to employment, and the subject’s intention not to participate) were eliminated during participation in the program. As a result, 44 participants finally participated: 23 in the experimental group and 21 in the control group (Table 1). The purpose and procedure of the study were explained to the participants before the start of the study, and they provided written consent, and compensation of USD 200.00 was given after participation in the mobile-based forest-therapy program was completed. This study was approved by the Institutional Review Board of Chungbuk National University (IRB No. CBNU-202303-HR-0042).

### 2.3. Study Location

**Figure 1:**
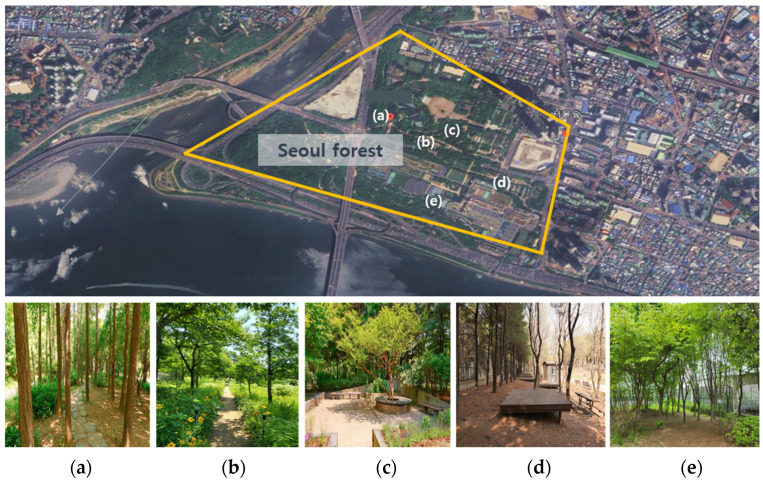
Mobile-based forest therapy program’s main activity place. (https://maps.app.goo.gl/VFxM758GNJZkHMUS9, accessed on 22 September 2023). (**a**) Ginkgo Forest (Meditation activity), (**b**) Forest Road (Five sense health walk), (**c**) Scent Garden (Emotional recognition activity), (**d**) Korean pine Tree Forest (Stretching), and (**e**) forest next to Butterfly Garden.

This study was conducted in ‘Seoul Forest’; in the capital city of Korea (Figure 1). Seoul Forest is an urban forest located in the city center, which is accessible to urban residents and is a representative natural space in the city center that many people visit. Seoul Forest opened in May 2006 and has a total area of 50 ha, with five themes: Culture and Arts Park, Natural Ecological Forest, Natural Experience Learning Center, Wetland Ecological Center, and Han River Waterfront Park. An herb garden is cultivated by residents, and ecological programs are provided to children and teenagers through wild animals, wetlands, and ecological parks. In addition, it is a healing space where you can enjoy nature as a space that provides forest experience programs for various ages. The main vegetation of the study target site included oak (*Quercus*), sargent cherry (*Prunus sargentii Rehder*), ginkgo (*Ginkgo biloba* L.), and korean red pine (*Pinus densiflora S. et Z*.). The average temperature in Seoul, which was the research site during the sixth session of this mobile-based forest-therapy program, was 19.9 °C.

### 2.4. Mobile-Based Forest-Therapy Program

**Figure 2:**
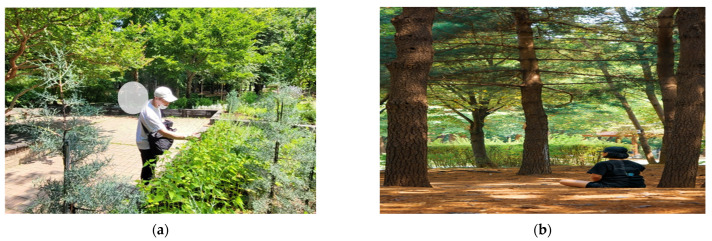

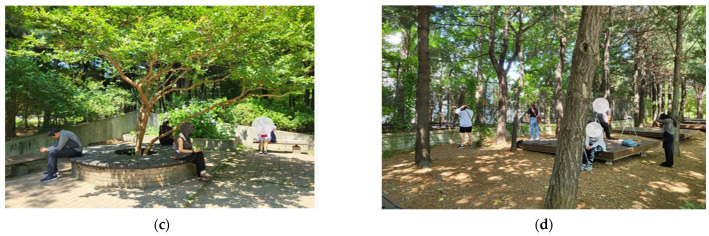
Activities of mobile-based forest-therapy program. (**a**) Walking on five senses of health (scent stimulation); (**b**) body scan (meditation); (**c**) finding emotions; and (**d**) stretching.

This study was conducted using Metri (App), a medical-based application without the guidance of a forest healing instructor. Metri is an IT service that conveniently helps patients to reserve, receive, and survey their prescription management, and chart this within the Department of Mental Health. It is used by psychiatrists in Seoul; therefore, it is highly useful for psychiatrists and patients. Five forest healing instructors participated in the development of a forest-therapy program to be applied to Metri. The forest-therapy instructor developed a forest-therapy program by combining exercise therapy and cognitive behavior therapy, which are highly likely to be applied clinically among non-pharmacological approaches to depression after subject analysis (Table 2). The forest-therapy program consisted of three stages. The first stage consisted of awareness of one’s body and mind; the second stage consisted of changes in one’s behavior; and the last three stages consisted of change. The forest-therapy program consisted of six programs at a time. Forest-therapy programs were conducted in the following order: forest greeting, stretching, five-sense health walking, meditation, sunlight sharing (finding emotions and wishes), and reading poems. Six programs were organized differently for each session, and forest-healing factors were experienced utilizing various spaces in Seoul Forest (Figure 2).

### 2.5. Study Procedure

**Figure 3:**
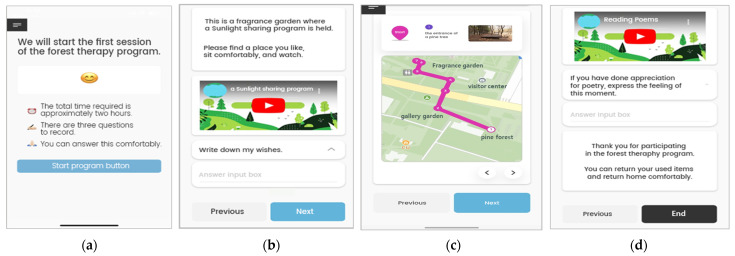
Guide to the mobile-based forest-therapy program of the ‘Metri’ application. (**a**) Forest- therapy program start screen; (**b**) Forest-therapy program video providing screen; (**c**) Forest- therapy Program Location Information Screen; (**d**) Forest-therapy program end screen.

The mobile-based forest-therapy program consisted of six sessions, starting on 28 April 2023, and ending on 10 June 2023. Participants were guided to download Metri in advance through Google Play and the App Store before participating in the mobile-based forest-therapy program. Before participating in the forest-therapy program, an orientation was conducted to explain the use of the application. On the day of the forest-therapy program, the researcher checked the attendance of the participants at the Seoul Forest meeting site and provided the goods necessary for participating in the program. The forest-therapy program began at 10 a.m. in Seoul Forest, and participants confirmed the prescription of the forest-therapy program for each session through its application and conducted the forest-therapy program on their own. The forest-therapy program took approximately 2 h, and the main usage method of Metri was as follows (Figure 3): (1) Run the application and press the start button of the forest-therapy program first (Figure 3a). (2) Watch the forest-therapy program image provided on the screen and press the next button at the bottom of the screen to proceed. For programs with questions, a simple answer is also provided (Figure 3b). (3) Guided maps can be used when moving places for forest-therapy programs, and the place can be checked through photos at the top (Figure 3c). (4) If you have participated in all the programs offered, you can exit the program by pressing the exit button at the bottom (Figure 3d).

### 2.6. Measurement

#### 2.6.1. MADRS (Montgomery–Asberg Depression Rating Scale)

It consists of 10 items developed by Montgomery and Asberg to evaluate the severity of depression [54]. The 7-point (0–6) Likert scale was used, and the total score ranged from 0 to 60. The evaluation score was 0–6: normal/symptom absent, 7–19: mild depression, 20–34: moderate depression, and 35–60: severe depression. In this study, the K-MADRS adapted and verified by Ahn et al. [55] was used, and its reliability was 0.96.

#### 2.6.2. PSQI (Pittsburgh Sleep Quality Index)

The Pittsburgh Sleep Quality Index is a subjective evaluation tool for sleep habits during the month. The PSQI, developed by Buysse et al. [56], consists of 18 questions related to sleep quality, sleep initiation incubation period, sleep time, sleep efficiency, sleep disorders, sleep medication use, and daytime dysfunction. The higher the PSQI score, the worse the sleep quality. The Korean version of the PSQI was used [57]. The Cronbach’s α for the PSQI-K was 0.84.

#### 2.6.3. PHQ-15 (Patient Health Questionnaire-15)

The Patient Health Questionnaire-15, developed by Kroenke et al. [58] was used to measure subjective somatic symptoms. The PHQ-15 consists of 15 questions and is a Likert 3-point scale: (0) not bothered at all; (1) slightly distressed; and (2) very distressed. The measurement results were divided into ≤5 points as low somatic symptoms, 6–10 point as medium somatic symptoms, and ≥11 points as high somatic symptoms. In this study, the Korean version scale standardized by Han et al. [59] was used, with Cronbach’s α = 0.83.

### 2.7. Analysis Method

The statistical analysis of this study was performed using SPSS 22.00 (SPSS, Chicago, IL, USA). Paired *t*-tests were used to compare the effects of depression improvement of participants between group-specific pre-tests and post-tests, and covariance analysis (ANCOVA) was conducted to compare the effects between groups. All statistical tests used *p*-value < 0.05 as a significant level.

## 3. Results

### 3.1. MADRS

**Figure 4:**
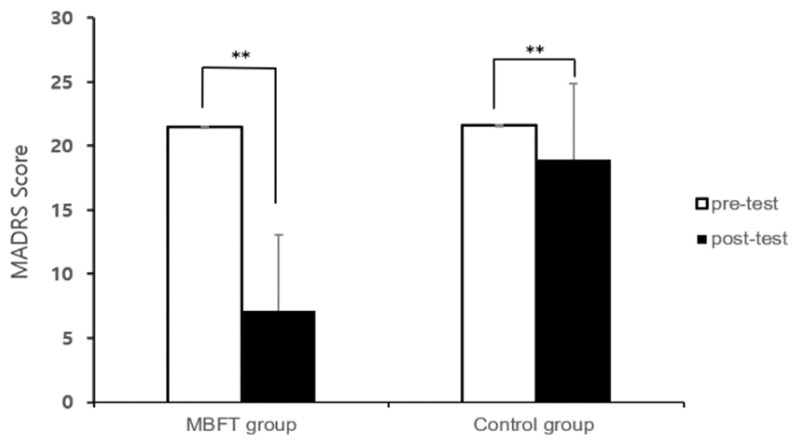
Results of measurement for pre- and post-test MADRS. Notes: MBFT: mobile-based forest therapy, **: *p* < 0.01.

The results of the paired *t*-test between pre- and post-test MADRS scores for each group are presented in Figure 4. As a result, it was shown that the mobile-based forest-therapy program had a significant effect on depressive symptoms in patients with depression (from 21.48 ± 4.05 to 7.13 ± 7.00, t = 10.139, *p* = 0.000). Also, there was a significant difference in the results of the control group’s MADRS (from 21.61 ± 5.81 to 18.95 ± 7.03, t = 3.630, *p* = 0.002).

Covariance analysis was conducted to analyze the effect of MADRS on score changes between the mobile-based forest-therapy program and control groups (Table 3). As a result of analyzing the difference in MADRS score changes between the two groups, patients with depression who participated in the mobile-based forest-therapy program showed a positive effect on MADRS compared to the control group (F = 50.074, *p* = 0.000).

### 3.2. PSQI

**Figure 5:**
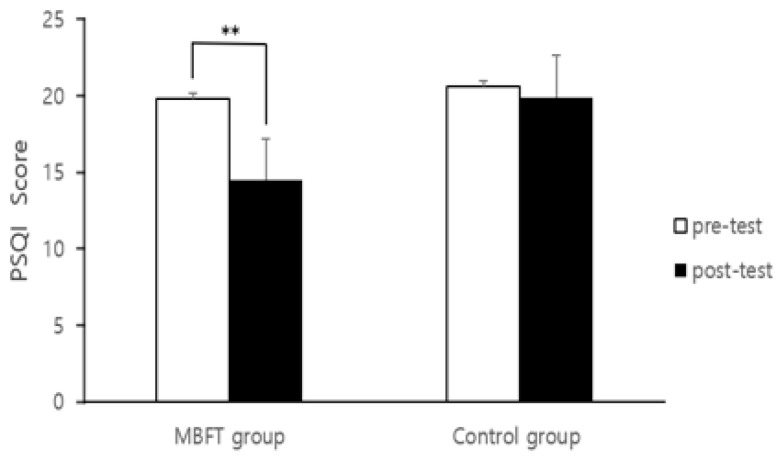
Results of measurement for pre- and post-test PSQI. Notes: MBFT: mobile-based forest therapy, **: *p* < 0.01.

The results of the paired *t*-test between the pre- and post-test PSQI scores for each group are shown in Figure 5. The results showed that the mobile-based forest-therapy program had a positive effect on the sleep quality of patients with depression. The degree of sleep difficulties of patients with depression who participated in the mobile-based forest-therapy program decreased, and the PSQI score decreased significantly (from 19.78 ± 7.69 to 14.48 ± 8.11, t = 3.301, *p* = 0.003). However, the control group did not show any difference in the sleep quality of depressed patients (from 20.57 ± 8.55 to 19.90 ± 8.58, t = 0.434, *p* = 0.669).

Covariance analysis was conducted to analyze the effect of PSQI on score changes between the mobile-based forest-therapy program and the control group (Table 4). The analysis of the difference in PSQI score concludes that it changes between the two groups and revealed that depressive patients who participated in the mobile-based forest-therapy program had a positive effect on PSQI compared to the control group (F = 5.945, *p* = 0.020).

### 3.3. PHQ-15

**Figure 6:**
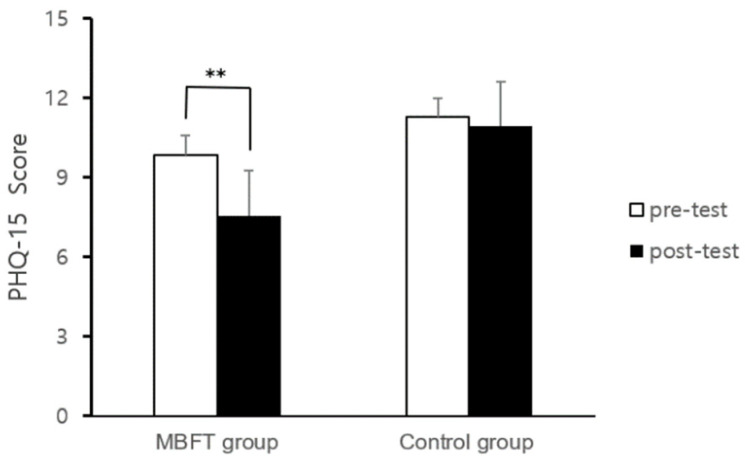
Results of measurement for pre- and post-test PHQ-15. Notes: MBFT: mobile-based forest therapy, **: *p* < 0.01.

The results of the paired *t*-test between the pre- and post-test PHQ-15 scores for each group are shown in Figure 6. The results showed that the mobile-based forest-therapy program had a significant effect on the subjective physical symptoms of depressed patients (from 9.87 ± 5.08 to 7.57 ± 5.03, t = 2.969, *p* = 0.007). However, the control group showed no significant differences in the PHQ-15 results (from 11.29 ± 5.82 to 10.95 ± 5.66, t = 0.360, *p* = 0.723).

Covariance analysis was conducted to analyze the effect of PHQ-15 on score changes between the mobile-based forest-therapy program and control group (Table 5). As a result of analyzing the difference in PHQ-15 score changes between the two groups, patients with depression who participated in the mobile-based forest-therapy program showed a significant effect on PHQ-15 compared to the control group (F = 4.442, *p* = 0.041).

## 4. Discussion

This study evaluated the effects of mobile-based forest-therapy programs in urban forests on depression, sleep quality, and physical symptoms in patients with depression. Mobile-based forest-therapy programs conducted in urban forests not only alleviate symptoms of depression but also improve sleep quality and physical symptoms. Many experimental studies have shown that forest-therapy programs provide psychological health benefits to participants. However, this research on the effectiveness of mobile-based forest-therapy programs for patients with mental illnesses, such as depression, is meaningful in that it was a new attempt.

First, participation in mobile-based forest-therapy programs has been shown to positively affect sleep quality in patients with depression. In contrast, there were no significant changes in daily life in the control group. These findings are consistent with previous studies indicating that participation in existing forest-therapy programs or natural activities can help alleviate insomnia [60,61,62,63]. Kim et al. [60] found that sleep duration increased after conducting a forest-therapy program for two days and one night for medical professionals suffering from excessive work stress caused by COVID-19. Kim et al. [61] participated in a forest-therapy program for cancer patients for 5 nights and 6 days and found that sleep time increased by about 30 min and sleep efficiency increased by 8%. It is important to improve the quality of sleep because sleep disorders, a major symptom of patients with depression and lethargy or fatigue, can cause difficulties in daily life. Providing forest-therapy programs for activities suitable for the subject suggests that it can have a positive effect on alleviating sleep-disorder symptoms in patients with depression.

Second, the experimental group that participated in the mobile-based forest-therapy program was found to have a positive effect on physical symptoms. However, no significant changes were observed in the control group. Since the physicalizing symptoms that appear in patients with depression are accompanied by emotional problems, activities that can relieve the depression and anxiety formed inside them are important. Prior research on changes in physicalizing symptoms in forest-therapy programs conducted for patients with depression remains insufficient. However, the meditation program applied in this study (e.g., walking meditation, breathing meditation, and pause meditation) was used to reflect on one’s mind or thoughts, and not to expand negative emotions. In addition, the sunshine-sharing program is believed to have affected depression or anxiety relief by having time to notice one’s feelings and thoughts about using emotion cards. This suggests that these stable changes in the body and mind led to an improvement in the physicalizing symptoms.

Finally, in the clinician’s evaluation using the MADRS for depression symptoms in depressed patients, both groups showed significant results in the pre–post comparison. In the mobile-based forest-therapy program group, the same trend was observed in previous studies showing that forest-therapy programs can alleviate depression. However, we found a significant effect in the control group, which is believed to be because the participants in the control group were treated with clinicians during the study period. Nevertheless, comparing the average values of the two groups showed that in the experimental group that received a mobile-based forest-therapy program, the measurement value decreased significantly from 21.48 ± 4.05 to 7.13 ± 7.00. However, in the control group, the measurement value changed from 21.61 ± 5.81 to 18.95 ± 7.03. These results were consistent with those of previous studies that showed that more positive effects could be achieved when hospital treatment and non-pharmaceutical treatment were combined. Pampallona et al. [64] reported that a meta-analysis study comparing the improvement of symptoms in patients who received drug treatment alone and those who received psychotherapy together showed a greater improvement when drug treatment and psychotherapy were combined.

Korea’s forest healing sector emphasizes the importance of providing medically linked forest-therapy programs to help people with depression continue to live healthy lives, and a therapeutic approach for better prognosis [65]. Although direct and indirect activities in nature have alleviated symptoms for the psychological, mental, and physiological health of depressed patients [46,47,48,49,50,51,60,61,62,63], data on the effectiveness of forest-therapy programs for mobile devices are in the early stages. The results of this study confirmed the potential for future use as the provision of mobile-based forest-therapy programs to patients with depression was found to have a positive effect on improving depression [30]. For depressed patients to increase their utilization of mobile-based forest-therapy programs, infrastructure construction and quality improvement are needed to enable the technical elements, components, and medical linkage of mental-health-related applications in the mobile market.

The number of smartphone mobile network subscribers worldwide is expected to exceed about 6.4 billion (as of 2022) [66], and Korea’s smartphone penetration rate is also known to be the worldwide highest at 95% [67]. With the emergence of the mobile healthcare concept, conditions have been created in the mobile market to fully utilize applications that provide mental health services. However, despite the low barriers to treatment compared with other treatments, away from problems such as location, time constraints, and social stigma [68,69], the continuity of using mobile applications related to mental health services has not been shown to last long [70,71]. Therefore, it is necessary to continue the interest of patients with depression by utilizing non-drug therapy that enables evidence-based clinical intervention rather than simply providing information. The use of mobile devices may differ in quality owing to the application of face-to-face nonpharmaceutical therapy. However, in real life, the consequences of an intervention can always be checked, which can play a role in continuing activities. Therefore, if the infrastructure for medically linked functions that can conveniently use the application, composition of prescriptions, and feedback on health conditions are supported, as a result utilization will be higher.

It is necessary to establish sufficient data to actively utilize mobile-based forest-therapy programs as a medical-welfare resource. Evaluating information on the condition of a patient with depression may prescribe a forest-therapy program that is suitable for the patient’s condition. When prescribing forest-therapy programs, psychiatrists and forest-healing instructors should cooperate to develop suitable programs for patients with depression. The information built through this will serve as a steppingstone for diagnosis and treatment plans for a patient’s condition by linking psychiatrists and patients through remote careers, counseling, and health monitoring. Establishing a policy proposal or legal system that can develop into a medically linked type, considering aspects such as the safety of patient data and personal information protection, will affect the growth of the mobile healthcare industry.

In the future, mobile-based forest-therapy programs are expected to be an alternative to new treatments for patients who are resistant to existing drug treatments or have difficulties using drug treatments. Forest-therapy programs based on urban forests around a living area can reduce the burden of the side effects of drugs and improve depression at an economical cost. If it is used as an adjuvant therapy for drug treatment, even after the development of a mental disease, cost savings can be expected by shortening the total treatment period. In addition, for patients with depression living in areas where medical benefits are difficult to receive, the foundation can be established in a better direction in terms of medical services. New avenues for mental health services could establish forest therapy as an alternative complementary medicine that complements the limitations of traditional clinical medicine for patients with depression.

Despite the potential use of mobile-based forest-therapy programs to improve symptoms in patients with depression, this study has limitations. First, this study is difficult to generalize because of the small number of study subjects. In addition, although the group was randomly assigned, the proportion of female participants was large, so the imbalance of the gender ratio within the group also served as a limitation. In addition, as the number of dropouts increases, it was found to be necessary to secure an appropriate number of people by fully considering demographic factors through a sufficient sampling period in future studies.

Second, it was difficult to confirm the specific progress of treatment in patients with depression. In this study, it was not confirmed whether the experimental and control groups received drug treatment and antidepressants, and whether they were involved in non-pharmaceutical treatment based on psychotherapy or cognitive behavior therapy. In future studies, it will be necessary to discuss the results by utilizing a checklist or diary on the treatment schedule and monitor drug use by each subject during the study period.

Third, an initial evaluation of the condition of patients with depression was not conducted. In this study, patients with mild depression were analyzed, but this may have affected the results depending on the history of each patient or their current depression. Future studies are needed to understand the patient’s condition through structured interviews with a psychiatrist.

Fourth, it was not possible to rule out the possibility of intervention in the frequency of contact with nature. This study was conducted in an urban forest located around a living area. However, both the experimental and control groups were unable to strictly control their daily lives despite the possibility of access to nature such as urban forests, nearby urban parks, and natural recreation forests in their daily lives. Future research will require additional understanding of the frequency of contact with nature, activities, and length of stay.

Fifth, the timing of mobile-based forest-therapy programs are limited. This study was conducted for six weeks and was limited to spring. There is a limit to discussing the effects of depression according to the sense of season because there are various healing resources experienced according to the sense of season. Future studies will be able to identify the impact on the forest environment or space suitable for patients with depression by comparing the effect analysis after each season’s forest-therapy program.

Six, the future effectiveness of the mobile-based forest-therapy program was not evaluated. It is necessary to check whether the six-week forest-therapy program was maintained.

Seventh, the impact of the application used in the mobile-based forest-therapy program was not identified. The existing forest-therapy program is guided by a forest healing instructor. It is difficult to conclude that forest-therapy programs using mobile devices are relatively familiar activities for participants. In this study, participants visited urban forests and relied entirely on applications. In addition, there would have been individual differences in the application’s ease of use and usability for smooth activities because not only are forest-therapy programs provided, but also the environment suitable for forest-therapy programs is set. Subjects who first visited Seoul Forest, which was the target site of this study, had difficulty finding the location of the forest-therapy program, even though they were guided with the use of maps and GPS. In addition, those who are older and those who are unfamiliar with mobile devices may be burdened with using the device. To facilitate user-centered participation in the program, it is also necessary to investigate differences due to external factors, such as the preference of mobile-based forest-therapy programs and difficulty in using applications.

Despite the limitations of this study, it is significant in that it has confirmed its potential as a non-pharmaceutical intervention by providing a systematic forest-therapy program to patients with depression through a mobile application and confirming its effectiveness in improving depressive symptoms. Mobile-based forest-therapy programs are expected to contribute to the diversification of intervention approaches to alleviate depression, not limited to time or place, and can be used as basic data for this purpose.

## 5. Conclusions

This study confirmed that depressive symptoms were significantly improved by applying a mobile-based forest-treatment program in patients with depression. The combination of natural activities and mobile application utilization experienced in urban forests near living areas is considered to have advantages such as accessibility, economic feasibility, reduced exposure to discrimination, patient empowerment, and data-based feedback. This is a very economical cost, which will make it easier for patients with depression to access treatment in their daily lives, reduce the burden of side effects, and provide a way to improve depression. Providing a variety of treatment approaches for patients with depression can have positive effects on preventing and treating depression.

## Figures and Tables

**Table 1:** Demographic characteristics of the mobile-based forest therapy and control groups.

Variable		Mobile-Based Forest-Therapy Group *N* = 23	Control Group *N* = 21
Sex	Male	6 (26.1)	8 (38.1)
	Female	17 (73.9)	13 (61.9)
Age	20s	5 (21.7)	5 (23.8)
	30s	9 (39.1)	10 (47.6)
	40s	6 (26.1)	4 (19.0)
	50s	2 (8.7)	-
	60s	1 (4.3)	2 (9.2)
Residence	Seoul	22 (95.7)	19 (90.5)
	Incheon/Gyeonggi	1 (4.3)	2 (9.5)
	Provinces	-	-
Academic ability	High school graduation	4 (17.4)	4 (19.0)
	University student	4 (17.4)	-
	Graduation from university	13 (56.5)	15 (71.4)
	Graduate or higher	2 (8.7)	2 (9.5)
Forest visit frequency	Every week	1 (4.3)	3 (14.3)
	1 times/month	1 (4.3)	3 (14.3)
	2 times/month	1 (4.3)	1 (4.8)
	1–2 times/4 months	9 (39.1)	2 (9.5)
	1–2 times/year	9 (39.1)	10 (47.6)
	Other Opinions	2 (8.7)	2 (9.5)

**Table 2:** Activity details of the mobile-based forest-therapy program.

Stage		1 Stage		2 Stage		3 Stage	
Objectives		Recognizing changes in one’s body and mind		Relax your mind and body by recognizing your emotions such as tension and anxiety		Recognizing stable changes in body and mind, embodying forest activities as a way of caring for oneself	
Session		1 session	2 session	3 session	4 session	5 session	6 session
Program name and activity	Forest greeting	Deciding my name using natural objects					
	Stretching	Stretching the whole body around the neck	Stretch the whole body around shoulders and arms	Stretch the whole body around the waist and side	Stretch the whole body around the wrist	Stretch the whole body around the hip and thigh	Stretch the whole body around the ankle
	Five-sense health walking	Walking in the right posture while looking at the forest scenery	Walking while listening to forest songs in the right posture	Wide walk	Walking with your hands behind your back, walking with the scent of the forest	Walking feeling the forest with your touch, walking barefoot	Walking freely
	Meditation	Breathing meditation	Breathing meditation	Body scan	Walking meditation	Stop and walk meditation	Wood meditation
	Sunlight sharing	Finding Emotions	Finding Emotions	Finding Wishes	Finding Wishes	Find Value	Tell the forest what I want to say
	Reading Poems (title)	Spring road	Mountain shadow dressed in spring clothes	Flowers and I	Wobbly flower	Resembling nature	Grain of jujube

**Table 3:** Results of covariance of MADRS.

Variable	Sum of Squares	df	Mean Square	F	*p*
MADRS					
Pre-test	838.394	1	838.394	27.965	0.000
Group	1501.198	1	1501.198	50.074	0.000 **
Error	1229.167	41	29.980		

Notes: **: *p* < 0.01.

**Table 4:** Results of covariance of PSQI.

Variable	Sum of Squares	df	Mean Square	F	*p*
PSQI					
Pre-test	1036.777	1	1036.777	22.577	0.000
Group	267.582	1	267.582	5.945	0.020 *
Error	1882.772	41	45.921		

Notes: *: *p* < 0.05.

**Table 5:** Results of covariance of PHQ-15.

Variable	Sum of Squares	df	Mean Square	F	*p*
PHQ-15					
Pre-test	633.805	1	633.805	46.173	0.000
Group	60.973	1	60.973	4.442	0.041 *
Error	562.800	41	13.727		

Notes: *: *p* < 0.05.

## Data Availability

No new data were created or analyzed in this study. Data sharing is not applicable to this article.
